# Comparison of statistical and machine learning models for healthcare cost data: a simulation study motivated by Oncology Care Model (OCM) data

**DOI:** 10.1186/s12913-020-05148-y

**Published:** 2020-04-25

**Authors:** Madhu Mazumdar, Jung-Yi Joyce Lin, Wei Zhang, Lihua Li, Mark Liu, Kavita Dharmarajan, Mark Sanderson, Luis Isola, Liangyuan Hu

**Affiliations:** 1 Institute for Healthcare Delivery Science, Department of Population Health Science and Policy, Icahn School of Medicine at Mount Sinai, New York, NY 10029 USA; 2 Tisch Cancer Institute, Mount Sinai Hospital, New York, NY 10029 USA; 3 Department of Mathematics and Statistics, University of Arkansas at Little Rock, Little Rock, AR 72204 USA; 4 Department of Radiation Oncology, Brookdale Department of Geriatrics and Palliative Medicine Mount Sinai Hospital, Icahn School of Medicine at Mount Sinai, New York, USA; 5 Department of Health System Design and Global Health, Icahn School of Medicine at Mount Sinai, New York, NY 10029 USA

**Keywords:** Oncology care model, Risk-adjustment model, Machine learning, Quantile regression, Generalized linear model

## Abstract

**Background:**

The Oncology Care Model (OCM) was developed as a payment model to encourage participating practices to provide better-quality care for cancer patients at a lower cost. The risk-adjustment model used in OCM is a Gamma generalized linear model (Gamma GLM) with log-link. The predicted value of expense for the episodes identified for our academic medical center (AMC), based on the model fitted to the national data, did not correlate well with our observed expense. This motivated us to fit the Gamma GLM to our AMC data and compare it with two other flexible modeling methods: Random Forest (RF) and Partially Linear Additive Quantile Regression (PLAQR). We also performed a simulation study to assess comparative performance of these methods and examined the impact of non-linearity and interaction effects, two understudied aspects in the field of cost prediction.

**Methods:**

The simulation was designed with an outcome of cost generated from four distributions: Gamma, Weibull, Log-normal with a heteroscedastic error term, and heavy-tailed. Simulation parameters both similar to and different from OCM data were considered. The performance metrics considered were the root mean square error (RMSE), mean absolute prediction error (MAPE), and cost accuracy (CA). Bootstrap resampling was utilized to estimate the operating characteristics of the performance metrics, which were described by boxplots.

**Results:**

RF attained the best performance with lowest RMSE, MAPE, and highest CA for most of the scenarios. When the models were misspecified, their performance was further differentiated. Model performance differed more for non-exponential than exponential outcome distributions.

**Conclusions:**

RF outperformed Gamma GLM and PLAQR in predicting overall and top decile costs. RF demonstrated improved prediction under various scenarios common in healthcare cost modeling. Additionally, RF did not require prespecification of outcome distribution, nonlinearity effect, or interaction terms. Therefore, RF appears to be the best tool to predict average cost. However, when the goal is to estimate extreme expenses, e.g., high cost episodes, the accuracy gained by RF versus its computational costs may need to be considered.

## Background

Scientific progress in oncology in the past two decades has led to new diagnostic tools and the development of novel targeted therapies. These discoveries have directly led to increased longevity in the setting of cancer. Therefore, many more patients are living with cancer as a chronic condition for longer periods of time and potentially accruing more healthcare costs over a lifetime [1]. It is estimated that the national cancer costs in the US will increase to $173 billion in 2020, which represents a 39% increase from 2010 [2], and is as such unsustainable for our health care system.

The Centers of Medicare & Medicaid Services (CMS) developed the Oncology Cancer Model (OCM) as an episode-based payment model to encourage participating practices to provide higher quality care at the same or lower cost for Medicare fee-for-service beneficiaries with cancer, in an effort to control healthcare costs related to cancer [3]. Each episode was defined as a 6-month period triggered by the receipt of outpatient non-topical chemotherapy or hormonal therapy for treatment of cancer. The OCM reimbursement model, initiated on July 1, 2016 for an anticipated 5 years (completing June 30, 2021) offers participating providers the potential to receive performance-based payments if they achieve certain quality outcomes and reduce their expenditures below a target price during the 6-month performance periods. A fundamental element of the OCM reimbursement model involves accurate target price-setting, as practices should only be held accountable for what they can manage. Thus, it is necessary to adjust for factors that drive expense over which a provider has no control (e.g. age, gender, case mix). CMS determines a target price based on historical utilization that is risk-adjusted, trended to the current performance period, and adjusted for new therapies that came into practice after model development. Determining a practice’s success relies on utilization of models that can reliably measure small savings improvements – on the order of 4 to 8% - above the background variation.

**Fig. 1:**
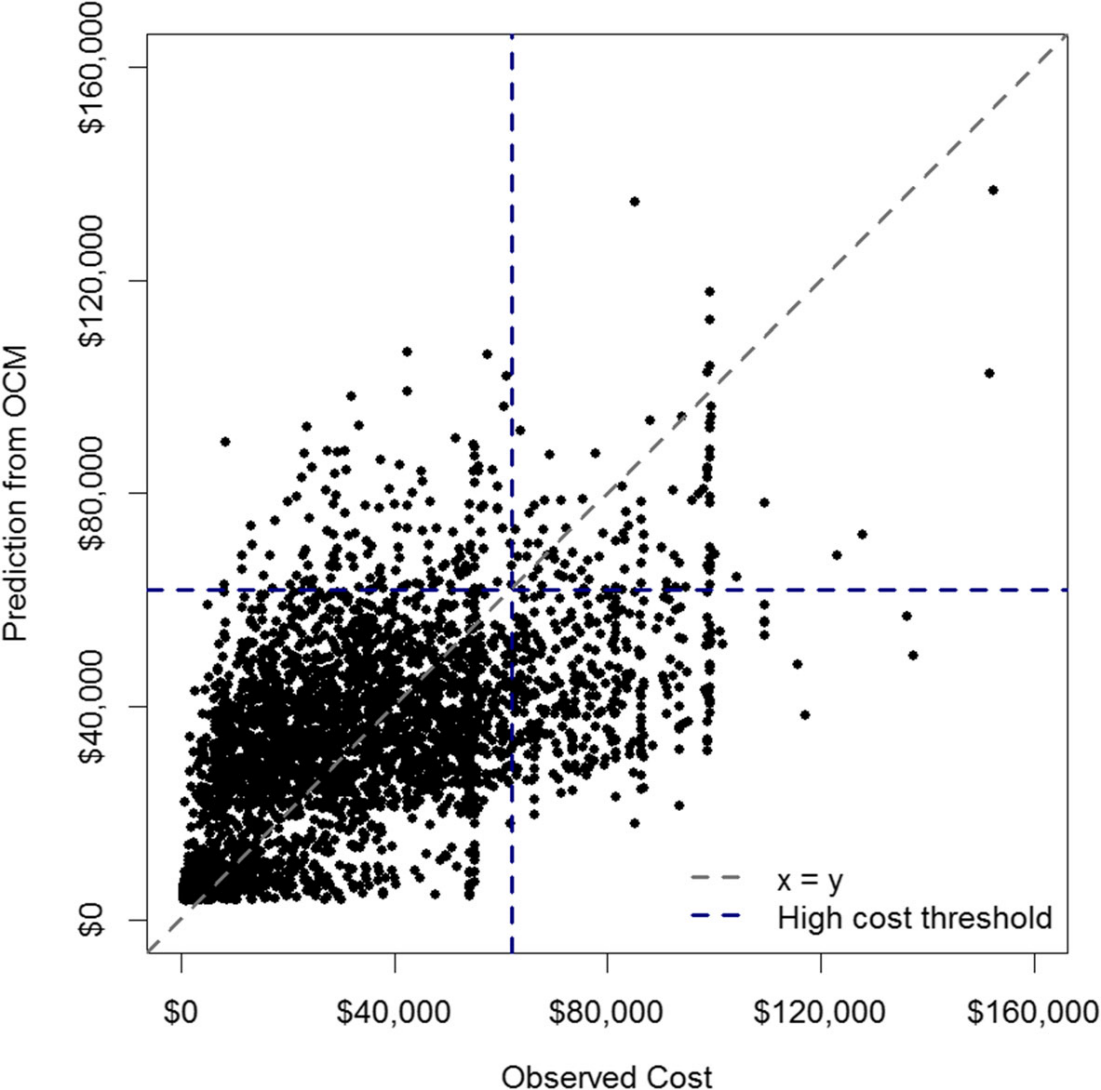
Observed versus expected expenses for the OCM model

Currently, the risk-adjustment model underlying OCM is a Gamma generalized linear model with log link (Gamma GLM). The model was originally developed using a national sample of over 2.7 million cancer episodes with 13 covariates [4]. OCM provided the original data on observed expenses, covariates, and predicted expenses back to the participating practices for the purpose of future planning. If the predicted expense correlated well with the observed expenses in the national sample, then individual practices would know to focus their performance improvement efforts in areas where actual expenses were meaningfully greater than expected. Unfortunately, the data for Mount Sinai OCM during the baseline period revealed that the OCM Gamma GLM model cost predictions did not correlate well with our observed cost data (see Fig. 1). This observation implies that Mount Sinai cancer care is inefficiently delivered, the OCM model is inaccurate, or both. To further understand potential reasons for this observed discrepancy, we were motivated to first closely examine the OCM data within a specific patient cohort for whom full cost data were available [5].

In this earlier work, we expected that residual variation (the difference between actual and expected expenses) should remain after risk adjustment, but were unclear whether what we observed in our data was valid. Several factors remain unadjusted for within OCM’s model such as the accurate capture of disease progression. While the OCM prostate cancer model includes a factor for castration-resistant prostate cancer, it does not further account for whether a given patient’s condition warrants particular 2nd line hormonal therapies [5]. These therapies’ costs alone exceed the upper limit for OCM prostate cancer episode price. Other OCM episodes, such as those for Breast Cancer, exhibit similar issues. CMS is working to improve the models’ factors to account for disease progression, so our next area of focus was to find whether there were better forms of predictive models for OCM episode data.

Healthcare cost data is usually skewed and heteroscedastic, meaning the variability of cost is unequal across the range of values of a second variable that predicts it. Gamma GLM is often used in practice to capture these features [6, 7]. However, Gamma GLM would have poor prediction accuracy when the underlying outcome distribution deviates somewhat from the Gamma distribution, or the log link is not the most suitable link [8–11]. For example, a Veteran Affairs study showed that prediction accuracy of a log link Gamma GLM was the lowest compared to alternative methods such as log normal, square root normal, and Gamma GLM with square-root link models [11]. Two other simulation studies also demonstrated that the coefficient estimates of the predictors from Gamma GLM were consistent but imprecise when the outcome did not follow Gamma distribution [9, 10]. Another general criticism of Gamma GLM is that it may not converge when the sample size is small to moderate [11]. Since it is impossible to know exactly if the outcome distribution belongs to a specific family of distribution, performance sensitivity at this level is not desirable. To this end, finding semi-parametric or nonparametric models, which are less sensitive to the specification of outcome distribution, is necessary. In addition, when covariates impact outcomes in a non-linear manner and when an interaction effect exists between covariates, ignoring these features in the specification or choice of model could affect model fit [12, 13]. Therefore, we searched for models that could incorporate these features without pre-specification of the functional form, or could approximate the relationship without needing its correct specification. We also examined the sensitivity of those results.

Two flexible modeling approaches, Partially Linear Additive Quantile Regression (PLAQR) and Random Forests (RF), emerged as strong candidates for comparison against the Gamma GLM model [6, 14]. The PLAQR model is a semi-parametric model, which allows the non-linear relationship of the outcome and covariates and makes no assumptions about the distribution of the error term. Thus, this approach has a greater flexibility than the Gamma GLM. The RF is a non-parametric approach with high prediction accuracy, capable of detecting interaction and nonlinearity without needing pre-specified functional form [15]. It has been promoted in clinical research, biomarker comparisons, and genomics studies [16–19]. However, the literature has only focused on comparing parametric cost prediction models; little is known about how these models perform on a relative basis [8, 10, 11].

We conducted a simulation study to compare the predictive accuracy of Gamma GLM, PLAQR, RF under a variety of scenarios regarding outcome distribution and model specification. In particular, we compared model performance in terms of the ability to predict average overall episode cost, and estimate the expense of the high-cost episodes. High-cost episodes were defined as episodes where costs exceeded the 90th percentile of the cost distribution. We also fit these models using Mount Sinai OCM payment data. Based on this information, we provide recommendations on which method was optimal for modeling healthcare cost data.

## Methods

### Overview of OCM data

Center for Medicare and Medicaid Innovations (CMMI) provided OCM payment data to each participating practice for episodes of care that were attributed to the practice. An episode was triggered by the receipt of a qualifying chemotherapy drug and included the sum of all Medicare facility and professional (Part A, B) and some pharmacy (Part D) expenses. The duration of the episode was 6 months from the trigger event. The data for the baseline period was provided at the episode level with actual and predicted expenditures. The 13 covariates used in the OCM risk adjustment model were 1) Age/Sex (10 categories), 2) Cancer type, 3) Chemotherapy drugs taken/administered during the episode (breast, bladder and prostate cancers only), 4) Receipt of cancer-related surgery, 5) Part D eligibility and dual eligibility for Medicare and Medicaid, 6) Receipt of radiation therapy, 7) Receipt of bone marrow transplant, 8) Clinical trial participation, 9) Comorbidities, 10) History of prior chemotherapy use, 11) Institutional status, 12) Episode length, and 13) Geographic location/Hospital Referral Region. Mount Sinai baseline data included 4205 episodes between January 1, 2012 and December 31, 2014, with the last episode end date on June 30, 2015. Computational details on how the baseline episode expenditure, target amount, and actual episode expenditure is available in the RTI International, 2017 and is constantly updated on the OCM website (https://innovation.cms.gov/initiatives/Oncology-Care/) [3, 4].

### Overview of statistical methods

Gamma GLM with log-link is widely used for healthcare cost prediction because it is amenable to the skewness and heteroscedasticity often observed in cost data [7, 20]. However, it has been criticized for its poor performance if the underlying outcome distribution deviates from Gamma distribution or the log link is not the most suitable [8–11]. Recently, semi-parametric and nonparametric models have been used in cost prediction due to their flexibility and ease of implementation. For example, Maidman et al. (2017) used PLAQR to predict future expense occurring at the upper (or lower) tail of the cost-distribution in the Medical Expenditure Panel Survey, a national survey that contains data from individuals’ medical providers and employers [6]. Wang et al. (2017) employed RF to stratify high-cost schizophrenia patients using administrative data [21]. However, use of these models is still limited. We briefly describe each method below and summarize their features in Table 1.

**Table 1:** Model characteristics for Gamma GLM, PLAQR, and Random Forest (RF) models

	Gamma GLM	PLAQR	Random Forest
Distribution assumption	Parametric	Semi-parametric	Nonparametric
Estimate	Mean	Quantile	Mean
Ability to model skewed outcome	Yes	Yes	Yes
Non-linear effect	Needs to be specified through model diagnostics	Needs to be specified (B-spline)	Data-driven detection; pre-specification not needed
Interaction effect	Needs to be specified through model diagnostics	Needs to be specified through model diagnostics	Data-driven detection; pre-specification not needed
Software	R, SAS, STATA	R	R, SAS

#### Gamma generalized linear model with log link (gamma GLM)

Generalized linear models (GLM) are an extension of linear regression with similar assumptions of independent and identically distributed data elements, a correct specification of the outcome distribution, and an appropriate link function (relationship between the covariates and the outcome). GLM is shown to be suitable for modeling the outcome distribution in the exponential family such as normal, lognormal, Weibull, and Gamma distribution. In other words, GLM can accommodate a variety of outcome characteristics such as skewness, heteroscedasticity, and being bounded or categorical. It is also able to handle a variety of links including inverse link, log link, and identity link. GLM assuming Gamma distribution (Gamma GLM) with a log link is appropriate for analyzing healthcare expense data, and is used for OCM cost modeling [4, 22, 23].

The Gamma GLM with log link is expressed as

1 $$ \mathbf{In}\left(\mathbf{E}\left(\mathbf{Y}\right)\right)=\mathbf{X}\boldsymbol{\upbeta }, \mathbf{Y}\sim \mathbf{Gamma}\left(\boldsymbol{\upkappa}, \boldsymbol{\uptheta} \right)\kern0.1em $$

where Y is the outcome, X denotes the covariate matrix, **β** is a vector of coefficients, and **κ**, **θ** are the location and rate parameters of the gamma distribution. While using this parametric model, model diagnostics were employed to find the functional form of non-linearity and interaction effects were pre-specified based on the subject matter knowledge. Models were then fitted with these specifications to make predictions. However, in simulation studies, B-spline basis functions (a nonparametric regression approach) were used for modeling the nonlinear relationship, and multiplicative interaction effects were specified. The latter was to match what was used in OCM. To stratify a high-cost episode using Gamma GLM, first the predicted cost for the episode from the fitted model was noted. It was labeled as a high-cost episode if the predicted cost was greater than a pre-defined threshold of the 90th percentile of the observed cost.

#### Partially linear additive quantile regression (PLAQR)

Quantile regression, which estimates the quantiles of the outcome conditional on the predictors, is a semiparametric regression model that can address the skewness and heteroscedasticity typical of cost data. Let ${Q}_{Y_i\mid {X}_i}\left(\tau \right)$ denote the *τ*^*th*^ (0 < *τ* < 1) quantile of outcome *Y*_*i*_ for the i^th^*e*pisode given the predictors *X*_*i*_, and *β*(*τ*) is the vector of coefficients for covariates *X*. The quantile regression can then be specified as.

2 $$ {\mathbf{Q}}_{\mathbf{Y}\boldsymbol{\upiota } \left|\mathbf{X}\boldsymbol{\upiota } \right.}\left(\boldsymbol{\uptau} \right)={\mathbf{X}}_{\mathbf{i}}\boldsymbol{\upbeta} \left(\boldsymbol{\uptau} \right)\kern0.1em $$

Maidman et al. (2017) extended this to partially linear additive quantile regression (PLAQR) by using the B-spline function to approximate the non-linear relationship [6]. The formula of PLAQR therefore changes to

3 $$ {\mathbf{Q}}_{\mathbf{Y}\boldsymbol{\upiota } \left|\mathbf{X}\boldsymbol{\upiota } \right.}\left(\boldsymbol{\uptau} \right)={\mathbf{V}}_{\mathbf{i}}\boldsymbol{\upbeta} \hbox{'}\left(\boldsymbol{\uptau} \right)+{\sum}_{\mathbf{k}=\mathbf{1}}^{\mathbf{q}}{\mathbf{g}}_{\mathbf{k}}\left({\mathbf{Z}}_{\mathbf{i}\mathbf{k}}\right) $$

where **β** ′ (**τ**) is a vector of the coefficients for covariates with linear effect, **V**_**i**_, and **g**_**k**_(**Z**_**ik**_) is the B-spline function for a q-vector covariates with non-linear effect, **Z**_**ik**_ (**k = 1,2,3,** …**,q;** ***i =*1**, **2**, …, ***n***).

Similar to Gamma GLM, we stratified and labeled an episode as a high-cost episode if the predicted cost from PLAQR was greater than the 90th percentile of the observed cost. To compare with other models, we fitted PLAQR in the original scale of the outcome and with ***τ*** = 50th percentile.

#### Random forests (RF)

The random forests (RF) is an ensemble learning technique that aggregates multiple decision trees. This aggregation drastically reduces variance compared to fitting a single decision tree. RF compares favorably with other frequently used nonparametric prediction models and can detect nonlinear covariate effects and interactions without any pre-specification [14, 24–26]. Here we consider Breiman’s original version of RF only, acknowledging that other variants exist [14].

The core idea of the RF algorithm is “bagging”, or bootstrap aggregating. In each bootstrap iteration, a single classification and regression tree (CART) is fit on the bootstrapped sample of the original data, excluding a proportion of the sample, referred to as “out-of-bag” (OOB) data. Then, predictions from each tree are obtained for the OOB data by dropping it down the tree. The average of OOB performance metrics can then be used to gauge the predictive performance of the entire ensemble. This value usually correlates well with the assessment of predictive performance we can get with either cross-validation or from a test set [27]. When growing individual trees in the ensemble, only a random subset of the covariates is used. This action saves run time but results in more diverse predictions from individual trees. The RF then bases predictions on the ensemble of these trees. For continuous outcomes, the predictions made by each tree are averaged. There are four tuning parameters for the RF algorithm, the number of candidate predictors used at each node, the number of trees to grow, the minimum size of terminal nodes and the maximum number of terminal nodes trees in the forest can have. Breiman (2001) proved that as the number of trees goes to infinity, there is a limit to the generalization error; therefore, increasing the number of trees does not cause the RF sequence to overfit the training data [24, 25]. However, an RF model can overfit the data if the average of fully grown trees results in too rich a model and incur unnecessary variance. Segal (2004) demonstrates small gains in performance by controlling the depths of the individual trees grown in the forests [28]. Empirical evidence shows that using full-grown trees seldom costs much [25]. All things considered, to avoid possible overfitting, we set the number of trees at a large number 1000, used the default values in the randomForest R package for the minimum size of terminal nodes and the maximum number of terminal nodes, allowing full-grown trees, and used cross validation to choose the optimal value of the number of candidate predictors, which is the number of covariates corresponding to the minimum OOB prediction error. In addition, we randomly split the OCM data into a training and a test set, and checked the test error to ensure that it is not substantially greater than the training error.

#### Criteria used for model comparison

We assessed overall accuracy of the predicted cost by the root-mean-squared error (RMSE), the mean absolute prediction error (MAPE), and cost accuracy (CA). These three metrics are commonly used to compare different cost-prediction models [11, 21, 29–31]. Let *y*_*i*_ and ${\hat{y}}_i$ denote the observed and predicted expenditure for episode *i* (*i* = 1, 2, …, *n*) respectively, RMSE, MAPE, and CA are defined as,

4 $$ \mathbf{RMSE}=\sqrt{\frac{\sum {\left({\hat{\mathbf{y}}}_{\mathbf{i}}-{\mathbf{y}}_{\mathbf{i}}\right)}^{\mathbf{2}}}{\mathbf{n}}} $$

5 $$ \mathbf{MAPE}=\frac{\sum \mid \hat{{\mathbf{y}}_{\mathbf{i}}}-{\mathbf{y}}_{\mathbf{i}}\mid }{\mathbf{n}} $$

and

6 $$ \mathbf{CA}=\frac{\mathbf{actual}\kern0.34em \mathbf{cost}\kern0.34em \mathbf{of}\kern0.17em \mathbf{predicted}\kern0.34em \mathbf{high}\hbox{-} \mathbf{cost}\kern0.34em \mathbf{episodes}}{\mathbf{actual}\kern0.34em \mathbf{cost}\kern0.34em \mathbf{of}\kern0.17em \mathbf{true}\kern0.34em \mathbf{high}\hbox{-} \mathbf{cost}\kern0.34em \mathbf{episodes}}\times \mathbf{100}\% $$

where CA is the proportion of high-cost dollars correctly predicted. The threshold of high cost is defined as the 90th percentile of the observed cost. Smaller values are desired for RMSE and MAPE and 100% is preferred for CA. A CA below one indicates that the model underpredicts the cost of episodes, whereas a CA above one means that the model overpredicts the cost of episodes.

A thousand bootstrap samples were generated, models were fit using the bootstrap samples, and boxplots of RMSE, MAPE, and CA are shown for model comparison.

### Simulation

#### Data generating process

Simulation was carried out using R 3.4.0 (Vienna, Austria) [32]. We used the characteristics of the OCM data as a guide when specifying outcome and covariate distributions and their parameters.

We generated 4205 observations (to match the amount of episodes from the Mount Sinai OCM data) and 10 covariates: ***x***_**1**_ and ***x***_**2**_ ~ Binomial (*n* = 5, *p* = 0.5) represented the ordinal variables; ***x***_**3**_ and ***x***_**4**_ ~ Binomial (*n* = 1, p = 0.5) the binary variables; ***x***_**5**_ and ***x***_**6**_ ~ Normal (0,1) the continuous variables with linear relationship with the outcome; ***x***_**7**_ ~ Binomial (*n* = 4,p = 0.5) the nominal variable, and ***x***_**8**_ ~ Normal(0,1), **z**_**1**_ ~ Uniform(0, 1), ***z***_**2**_ ~ Uniform (− 1,1) as continuous variables with non-linear relationship with the outcome.

We then generated **y**^′^ as a function of the covariates ***X***,

7 $$ {\mathbf{y}}^{\prime }=\mathbf{11.05}+\mathbf{0.05}{\mathbf{x}}_{\mathbf{1}}-\mathbf{0.08}{\mathbf{x}}_{\mathbf{2}}+\mathbf{0.2}\mathbf{5}{\mathbf{x}}_{\mathbf{3}}+\mathbf{0.15}{\mathbf{x}}_{\mathbf{4}}-\mathbf{0.02}{\mathbf{x}}_{\mathbf{5}}-\mathbf{0.002}{\mathbf{x}}_{\mathbf{6}}-\mathbf{2.3}{\mathbf{x}}_{\mathbf{71}}-\mathbf{1.3}{\mathbf{x}}_{\mathbf{72}}-\mathbf{0.4}{\mathbf{x}}_{\mathbf{73}}-\mathbf{0.1}{\mathbf{x}}_{\mathbf{74}}-\mathbf{0.75}{{\mathbf{z}}_{\mathbf{1}}}^{\mathbf{2}}+\mathbf{0.15}{{\mathbf{z}}_{\mathbf{2}}}^{\mathbf{3}}-\mathbf{0.35}\frac{{{\mathbf{x}}_{\mathbf{8}}}^{\mathbf{2}}}{\mathbf{1}+{{\mathbf{x}}_{\mathbf{8}}}^{\mathbf{2}}}+\mathbf{0.2}{\mathbf{x}}_{\mathbf{3}}{\mathbf{x}}_{\mathbf{5}}, $$

where **x**_**71**_**,x**_**72**_**,x**_**73**_**,x**_**74**_ were dummy variables for **x**_**7**_ and coefficients were guided by the Gamma GLM fit to the Mount Sinai OCM data and chosen to match their summary statistics (e.g. mean, median, and 90th percentile of the simulated cost). Although we generated the data following the guidance of OCM data and kept a few settings close to it, we also wanted to explore how the model performed under different distributions. Therefore, for the outcome **y**, we consider distributions common in the health economics and health services research literature [10, 33]. Distributions considered from exponential family were Gamma and Weibull distribution; from the non-exponential family, we generated a log-normal distribution with a heteroscedastic error term and a heavy-tailed distribution mimicking a mixture of normal distributions. These distributions have different skewness (measure of asymmetry of a frequency distribution) and different kurtosis (the sharpness of the peak of a frequency-distribution curve). All are skewed to the right and take only non-negative values.

For the outcome distribution generation, the following specifications were used. For the Weibull distribution, we generated **y** ~ Weibull (shape = 8, scale = 1.12 **y**′); for the Gamma distribution **y** ~ Gamma (**shape = 48,rate = 48**/**y**′).

To construct the heteroscedastic log-normal data, we considered **y = y**^′^**+ ε × v**(**x**)**,ε~N**(**0,0.1**); **v**(**x**) **= 0.5 + x**_**1**_.

To generate skewed cost data with a heavy tail, we considered a mixture of two log-normal distributions, **y = y**^′^**+ 0.9*ε***_**1**_**+ 0.1*ε***_**2**_**,** where ***ε***_**1**_**~N**(**0**, **1**) and ***ε***_**2**_**~*N***(**0**, **2**).

**Fig. 2:**
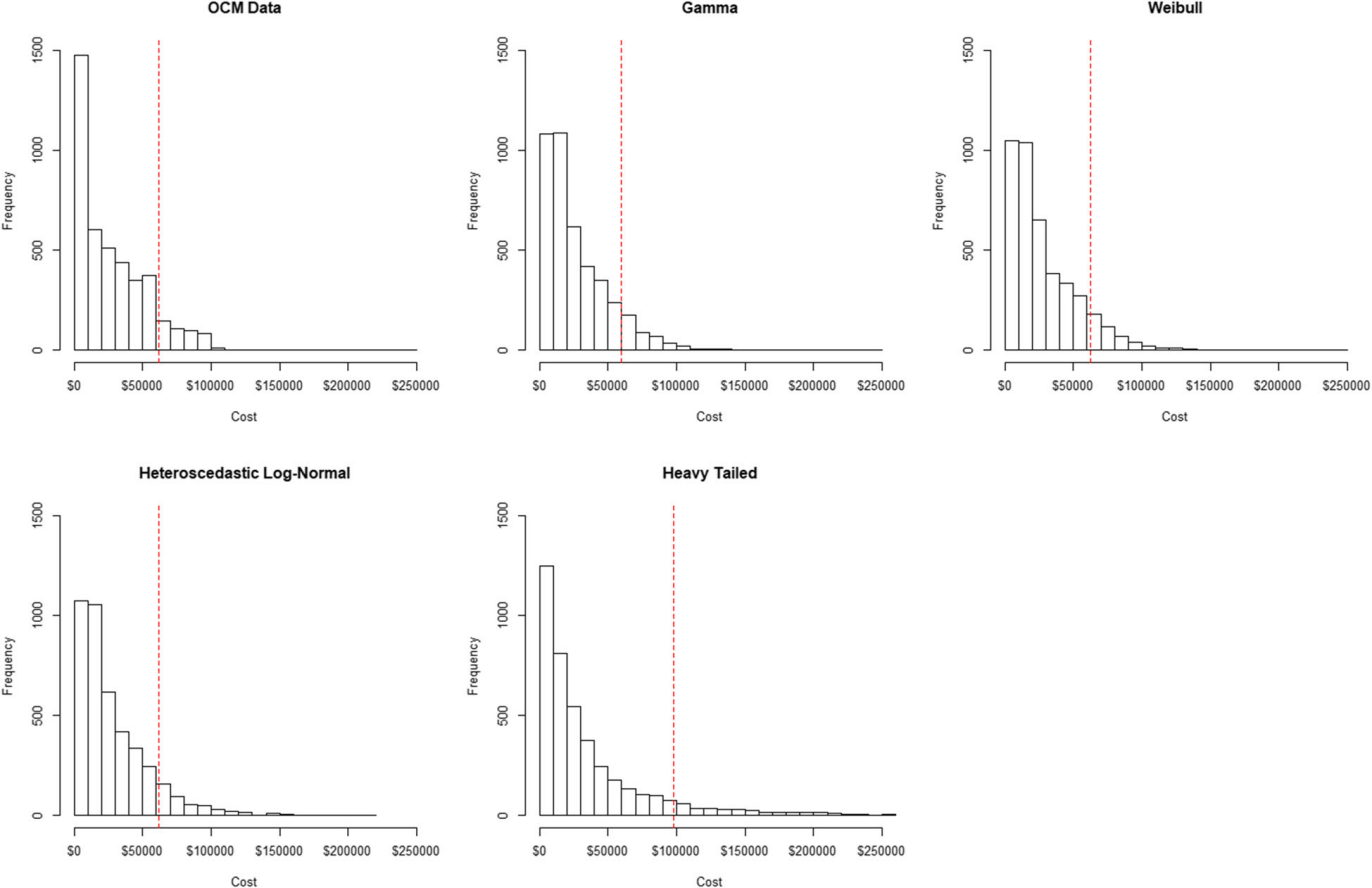
Actual expenses in Mount Sinai OCM data versus simulated data. Simulated data included distribution from exponential (Gamma and Weibull) and non-exponential families (Heteroscedastic log-normal and heavy-tailed). The red vertical line indicates the 90th percentile for each distribution

In Table 2, we summarize the final parameter estimates of the distribution achieved. Figure 2 showed the density plots of OCM data and the four simulated outcome data distributions. Our goal was to match the mean, median and the 90th percentile of some distributions to the OCM payment data, to find the best method for cost data structures observed in the OCM data.

**Table 2:** Summary statistics for actual expenses from OCM data at Mount Sinai (*N* = 4205) and simulated distributions

	Mean	Median	90th percentile	Skewness	Kurtosis
OCM data	$27,329	$20,552	$61,885	1.0	0.6
Gamma	$27,023	$19,378	$59,058	1.5	2.3
Weibull	$28,488	$20,498	$63,108	1.5	2.4
Heteroscedastic log-normal	$28,653	$19,700	$62,159	2.8	21.5
Heavy tail	$39,368	$18,844	$91,454	5.7	53.2

#### Model misspecification

To compare the performance of the three methods when the model was misspecified, we considered scenarios where models are correctly specified with nonlinearity and interaction effects. Misspecification was simulated by keeping only the linear forms of the covariates and by removing the interaction effect in the model. This kind of mis-specification was only expected to affect GLM and PLAQR, since RF requires only specification of the main effects (minimum user input) and implicitly identifies non-linearity and interactions.

### Data analysis for Mount-Sinai OCM data

We then applied these methods (Gamma GLM, RF, PLAQR) to the Mount Sinai OCM data. We used the same 13 covariates as in the OCM model fit to the national data. All analyses were done using R 3.4.0 (Vienna, Austria) [32].

## Results

### Results from the simulation study

**Fig. 3:**
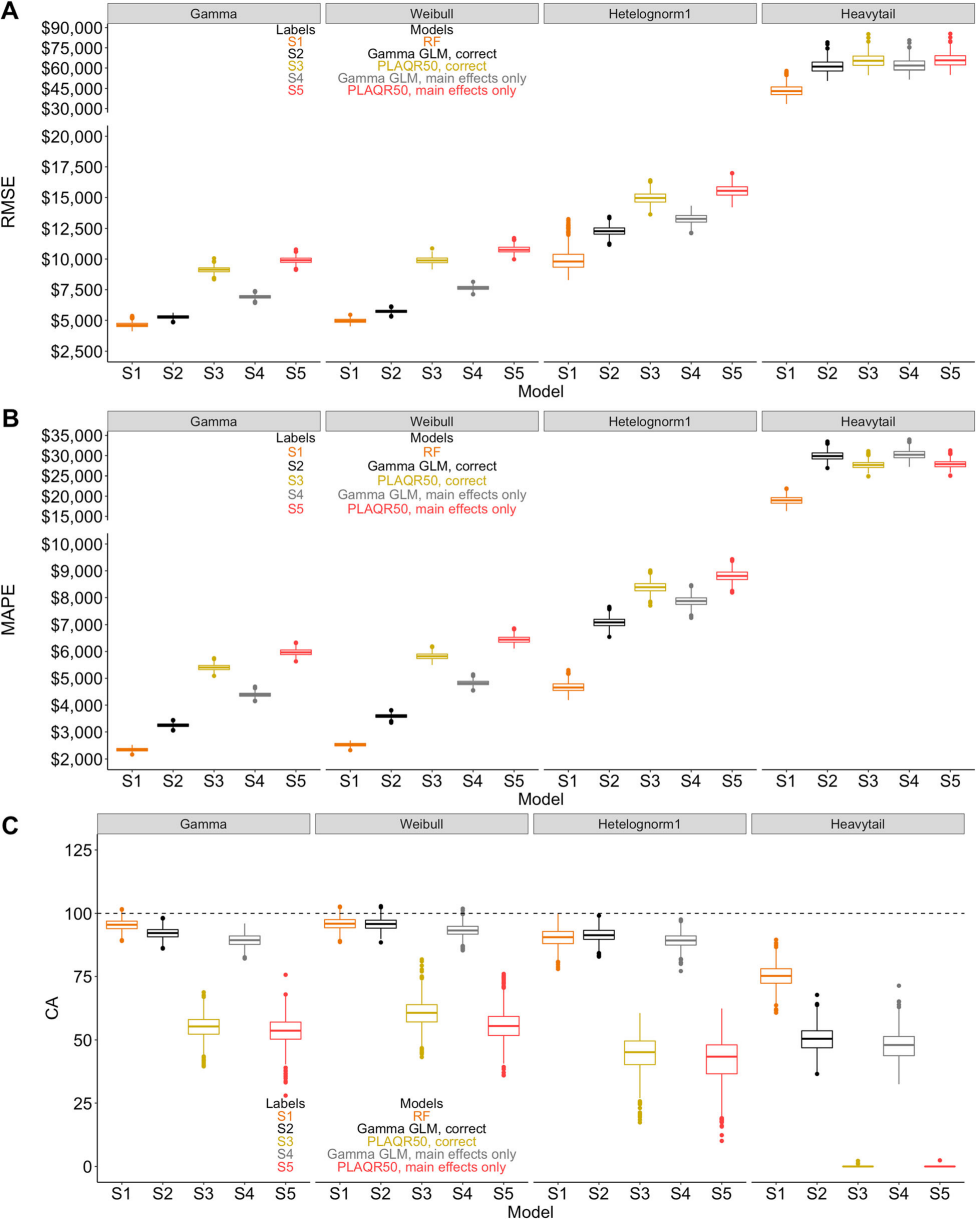
**a**-**c**. Accuracy of predicted cost in correctly and incorrectly specified models for simulated data. 3A: RMSE, 3B: MAPE, 3C: CA; Performance metrices estimated from 1000 bootstrapped samples. Gamma GLM, PLAQR estimating 50th percentile on original scale, and RF with original scale of cost are shown

The comparative performance metrics of all three methods under correct and misspecified models are shown in Fig. 3a, b, and c. Under correct model specification when the outcome distribution was from the non-exponential family (heteroscedastic log-normal or heavy tail distribution), RF had the best performance by all three metrics. The difference between RF and correctly specified Gamma GLM was small for Gamma and Weibull outcome distributions. Within each distribution, under conditions of model misspecification, PLAQR and Gamma GLM’s performance worsened. This situation was more pronounced under the non-exponential models of log-normal with heteroscedasticity and heavy tail distribution.

### Results from analysis of OCM data

**Fig. 4:**
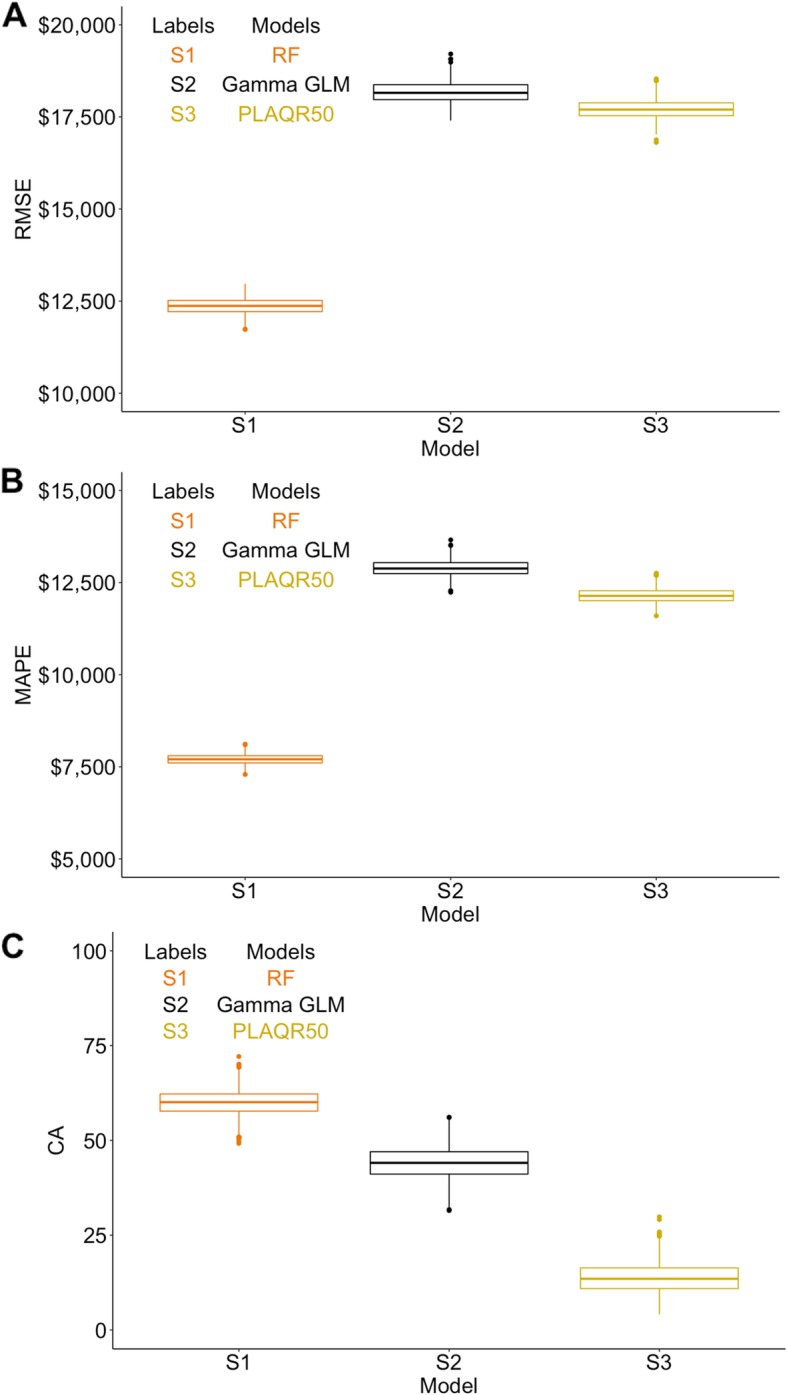
**a**-**c**. Accuracy of predicted cost in correctly specified models for Mount Sinai OCM data. 4A: RMSE, 4B: MAPE, 4C: CA; Performance metrics estimated from the 1000 bootstrapped samples. Results using Gamma GLM, PLAQR estimating 50th percentile on original scale, and RF with original scale of cost are shown

The comparative performance metrics for all three methods are shown in Fig. 4 a, b, and c. RF performed the best in the Mount Sinai OCM data setting. Table 3 shows RMSEs and MAPE for different percentiles of costs. The RF model consistently produced the smallest errors across all percentiles, while the 90th -100th and 80th–90th percentiles had the largest prediction errors. Table S1 in Supplemental Materials shows that RF had the smallest RMSE on the test data among the three methods.

**Table 3:** RMSE and MAPE of Random Forest (RF), Gamma GLM and PLAQR implemented on OCM data in different percentiles

RMSE		0 - 20th	20 - 40th	40 - 60th	60 - 80th	80th - 90th	90th - 100th
	RF	$3661	$8444	$8246	$6583	$11,174	$17,411
	Gamma GLM	$5881	$16,548	$18,291	$15.246	$21,446	$33,276
	PLAQR	$5312	$15,333	$14,826	$12,715	$21,995	$37,253
MAPE	RF	$2436	$6048	$6712	$5008	$9044	$15,406
	Gamma GLM	$4098	$11,033	$14,362	$11,501	$18,477	$29,234
	PLAQR	$2668	$10,749	$11,933	$9760	$18,620	$34,102

## Discussion

In our simulation study and data application, RF outperformed other models for predicting average cost and identifying high-cost episodes in most scenarios. The superior performance of RF was more pronounced when the model was misspecified. Misspecification of non-linear effects and interaction terms in models is common because knowing the exact outcome distribution or the exact relationship within and between the covariates is generally impossible. Therefore, this data-driven characteristic of RF makes it suitable for modeling health care costs, since non-linearity and interaction effects are expected. We also compared the performance of models in smaller samples (*n* = 200 and *n* = 500) (see Supplementary Figs. S1A-S1D). RF performance was more sensitive to sample size when the outcome distribution was in the exponential family. With a large sample, the advantage of RF was more pronounced even compared to correctly specified Gamma GLM and PLAQR. With a small sample, it was more advantageous than Gamma GLM and the PLAQR main effects-only model.

When the models were compared across the various outcome distributions, all behaved in a similar manner when the distributions belonged to the exponential family (Gamma and Weibull). However, all methods were sensitive (with RF being least sensitive) to outliers in the cost data, which were more frequently generated in models of the non-exponential family (Log-normal with heteroscedastic error term and Mixture normal [heavy-tailed]). Therefore, RF is the better method for cost distribution with outliers. When the cost distribution is in the exponential family, RF and Gamma GLM has similar accuracy in classification. However, RF requires longer computational time. Therefore, if the purpose of a study is to identify the extremes, e.g., the high-cost episodes in our case, the classification accuracy gained by RF must be weighed versus the increased computational time.

A concern for Academic Medical Centers (AMCs) such as Mount Sinai is that their patients are sicker than average, and the associated costs for their care tend to be the highest [34]. However, nearly all models underpredict high-cost episodes, which does not favor AMCs [35]. As a result, comparing AMCs to the entire country, as in the OCM risk model, introduces some bias. Our study showed that RF improved overall accuracy of cost prediction compared to Gamma GLM and PLAQR, and improved accuracy among high-cost episodes. Thus, using RF could reduce bias against AMCs when their cost of care is compared to a national sample.

All participants in the OCM are challenged by variance. It remains unclear whether risk adjustment models can accurately detect changes in average episode expenses enough to drive incentive payments. In best-case scenarios, interventions that reduce episode costs, such as lowering admissions and emergency room visits, may reduce average episode costs by 10%. For Mount Sinai’s episode distributions that corresponds to identifying changes on the order of $2200. This is a measurement challenge given that even our correctly specified models applied against simulation data had accuracy metrics well above 10% of the mean. This is further compounded by the real-world challenge of small sample sizes: only very large practices may have enough episodes during a performance period to detect a 10% episode cost change. How to develop models that can reliably and accurately detect small changes in mean cost based on sample sizes of 50–200 episodes requires further study.

## Conclusions

RF outperformed Gamma GLM and PLAQR in predicting overall and top decile costs. RF demonstrated improved prediction under most scenarios in this simulation study. Additionally, RF did not require prespecification of outcome distribution, nonlinearity effect, or interaction terms. Therefore, RF appears to be the best tool to predict average cost. However, when the goal is to estimate extreme expenses, e.g., high cost episodes, we may consider the accuracy gained by RF versus its computational costs.

## Supplementary information

**Additional file 1: Figure S1A-D**: RMSEs estimated from the five models with varied sample sizes. S1A: Gamma distribution, S1B: Weibull distribution, S1C: Heteroscedastic log-normal distribution, S1D: Heavy-tailed distribution. Results were plotted based on 1000 bootstrapped samples except for correctly specified and main effects-only Gamma GLM for heavy-tailed distribution (*N* = 200). Results using Gamma GLM with correct specification of interaction and non-linear terms for 200 samples, plotted based on the 286 converged models. Results using Gamma GLM with main effects only for the same scenario were plotted based on the 680 converged models. Results using Gamma GLM, PLAQR estimating 50th percentile on original scale, and RF with original scale of cost are shown.

## Data Availability

Codes for data simulation are available at GitHub (simulation codes). The simulated data could be generated by running those codes. Deidentified Oncology Care Model data for Mount Sinai Hospital would be available from the corresponding author.

## Abbreviations

OCM: Oncology Care Model (OCM)
Gamma GLM: Gamma generalized linear model
RF: Random Forest
PLAQR: Partially Linear Additive Quantile Regression
RMSE: root mean square error
MAPE: Mean absolute prediction error
CA: Cost accuracy
CMS: Centers of Medicare & Medicaid Services (CMS)
CMMI: Center for Medicare and Medicaid Innovations
SD: Standard deviation
CI: Confidence interval
CART: Classification and regression tree
OOB: Out of bag
AMCs: academic medical centers

## References

[1] Siegel RL, Miller KD, Jemal A (2019). Cancer statistics, 2019. CA Cancer J Clin.

[2] Mariotto AB, Yabroff KR, Shao Y, Feuer EJ, Brown ML (2011). Projections of the cost of cancer care in the United States: 2010-2020. J Natl Cancer Inst.

[3] Oncology Care Model: Centers for Medicare & Medicaid Services (CMS); 2019 [Available from: https://innovation.cms.gov/initiatives/oncology-care/]. Accessed 16 Dec 2019.

[4] RTI International ARC (2017). OCM performance-based payment methodology. In: Services CfMM.

[5] Ennis RD, Parikh AB, Sanderson M, Liu M, Isola L. Interpreting Oncology Care Model Data to Drive Value-Based Care: A Prostate Cancer Analysis. J Oncol Pract. 2019;15(3):e238–e46. 10.1200/JOP.18.00336 30742551

[6] Maidman A, Wang L. New semiparametric method for predicting high-cost patients. Biometrics. 2017:1104–11. 10.1111/biom.12834 29228454

[7] Barber J, Thompson S. Multiple regression of cost data: use of generalised linear models. J Health Serv Res Pol. 2004;9(4):197–204. 10.1258/1355819042250249 15509405

[8] Mihaylova B, Briggs A, O'Hagan A, Thompson SG. Review of statistical methods for analysing healthcare resources and costs. Health Econ. 2011;20(8):897–916. 10.1002/hec.1653 PMC3470917 20799344

[9] Manning WG, Mullahy J (2001). Estimating log models: to transform or not to transform?. J Health Econ.

[10] Manning WG, Basu A, Mullahy J (2005). Generalized modeling approaches to risk adjustment of skewed outcomes data. J Health Econ.

[11] Montez-Rath M, Christiansen CL, Ettner SL, Loveland S, Rosen AK (2006). Performance of statistical models to predict mental health and substance abuse cost. BMC Med Res Methodol.

[12] Braumoeller BF (2004). Hypothesis testing and multiplicative interaction terms. Int Organ.

[13] Harrell FE, Lee KL, Mark DB (1996). Multivariable prognostic models: issues in developing models, evaluating assumptions and adequacy, and measuring and reducing errors. Stat Med.

[14] Breiman L (2001). Random forests. Mach Learn.

[15] Ryo M, Rillig MC. Statistically reinforced machine learning for nonlinear patterns and variable interactions. Ecosphere. 2017;8(11):e01976.

[16] Churpek MM, Yuen TC, Winslow C, Meltzer DO, Kattan MW, Edelson DP (2016). Multicenter comparison of machine learning methods and conventional regression for predicting clinical deterioration on the wards. Crit Care Med.

[17] Nguyen T (2017). Using random Forest model for risk prediction of hospitalization and Rehospitalization associated with chronic obstructive pulmonary disease [thesis or dissertation].

[18] Schulz A, Zoller D, Nickels S, Beutel ME, Blettner M, Wild PS (2017). Simulation of complex data structures for planning of studies with focus on biomarker comparison. BMC Med Res Methodol.

[19] Chen X, Ishwaran H (2012). Random forests for genomic data analysis. Genomics..

[20] Ng VKY, Cribbie RA (2017). Using the gamma generalized linear model for modeling continuous, skewed and Heteroscedastic outcomes in psychology. Curr Psychol.

[21] Wang Y, Iyengar V, Hu J, Kho D, Falconer E, Docherty JP (2017). Predicting future high-cost schizophrenia patients using high-dimensional administrative data. Front Psychiatry.

[22] Dodd S, Bassi A, Bodger K, Williamson P (2006). A comparison of multivariable regression models to analyse cost data. J Eval Clin Pract.

[23] Deb P, Norton EC (2018). Modeling health care expenditures and use. Annu Rev Public Health.

[24] Breiman L (2001). Using iterated bagging to debias regressions. Mach Learn.

[25] Hastie T, Tibshirani R, Friedman JH (2009). The elements of statistical learning : data mining, inference, and prediction.

[26] Murphy KP (2012). Machine learning: a probabilistic perspective. Machine Learning: A Probabilistic Perspective.

[27] Kuhn M, Johnson K (2013). Applied predictive modeling.

[28] Segal M. Machine Learning Benchmarks and Random Forest Regression. Technical report. eScholarship Repository: University of California; 2004. [Available from: http://repositories.edlib.org/cbmb/bench_rf_regn]..

[29] Meenan RT, Goodman MJ, Fishman PA, Hornbrook MC, O'Keeffe-Rosetti MC, Bachman DJ (2003). Using risk-adjustment models to identify high-cost risks. Med Care.

[30] Buntin MB, Zaslavsky AM (2004). Too much ado about two-part models and transformation? Comparing methods of modeling Medicare expenditures. J Health Econ.

[31] Tamang S, Milstein A, Sorensen HT, Pedersen L, Mackey L, Betterton JR (2017). Predicting patient 'cost blooms' in Denmark: a longitudinal population-based study. BMJ Open.

[32] Development R. Core team. R: a language and environment for statistical computing. Vienna, Austria: R Foundation for Statistical. Computing. 2017.

[33] Malehi AS, Pourmotahari F, Angali KA (2015). Statistical models for the analysis of skewed healthcare cost data: a simulation study. Heal Econ Rev.

[34] Slavin PL (2011). Commentary: health care reform and the finances of academic medical centers. Acad Med.

[35] Hileman G, Steele S. Accuracy of Claims-Based Risk Scoring Models. Society of Actuaries; 2016.

