# Supplementary material for: Comparison of statistical and machine learning models for healthcare cost data: a simulation study motivated by Oncology Care Model (OCM) data
Source: BMC Health Serv Res. 2020 Apr 25;20:350. doi: 10.1186/s12913-020-05148-y (PMC7183716; doi:10.1186/s12913-020-05148-y)
Supplement: Supplementary file 1 — Additional file 1: Figure S1A-D: RMSEs estimated from the five models with varied sample sizes. S1A: Gamma distribution, S1B: Weibull distribution, S1C: Heteroscedastic log-normal distribution, S1D: Heavy-tailed distribution. Results were plotted based on 1000 bootstrapped samples except for correctly specified and main effects-only Gamma GLM for heavy-tailed distribution (N = 200). Results using Gamma GLM with correct specification of interaction and non-linear terms for 200 samples, plotted based on the 286 converged models. Results using Gamma GLM with main effects only for the same scenario were plotted based on the 680 converged models. Results using Gamma GLM, PLAQR estimating 50th percentile on original scale, and RF with original scale of cost are shown. [file 12913_2020_5148_MOESM1_ESM.docx]

Supplemental Materials for “Comparison of Statistical and Machine Learning Models for Healthcare Cost Data: A Simulation Study Motivated by Oncology Care Model (OCM) Data” by

Madhu Mazumdar^1,3^, Jung-Yi (Joyce) Lin^1,3^, Wei Zhang^2^, Lihua Li ^1,3^, Mark Liu^3^, Kavita Dharmarajan^4^, Mark Sanderson^5^, Luis Isola^3^, Liangyuan Hu*^1,3^

***Section 1. Small sample simulation***

We fitted Gamma GLM and PLAQR under the *correct* model specification (for best performance) and also with main effects only (as commonly implemented in practice), and compared them with RF using the RMSEs, computed from 1000 replications. The results are shown in the figures below. When the sample size was 200 and distribution was heavy tail, Gamma GLM with correct specification converged in only 286 out of 1000 bootstraps and Gamma GLM with main effects converged in 680 out of 1000 bootstraps. The boxplots of RMSE from Gamma GLMs with correct specification and main effects only under that scenario were plotted based on the converged Gamma GLMs. The variance of RMSE increased as the sample size decreased, indicating diminished model stability with smaller sample size (see Figures S1A, S1B, S1C, and S1D). The performances of RF, Gamma GLM, and PLAQR did not vary much with the sample size under heteroscedastic log-normal and heavy-tailed distribution. RF was the top performer followed by Gamma GLM and PLAQR (see Figure S1C and Figure S1D). However, for outcome distributions from the exponential family, specifically Gamma and Weibull distributions, the RMSE of RF went up as the sample size went down, whereas the RMSE of PLAQR and Gamma GLM roughly stayed the same, rendering Gamma GLM, if correctly specified, as the best model followed by RF and PLAQR (see Figure S1A and Figure S1B). However, if only main effects were included in the model, RF had the best performance of all three methods except for the setting where the sample size was 200 and the outcome followed gamma distribution, in which case Gamma GLM had the lowest RMSE (see Figures S1A, S1B, S1C, and S1D). Overall, the advantage of random forests is more pronounced when the sample size is large even compared to best possibly fitted Gamma GLM and PLAQR; or compared to Gamma GLM and PLAQR main effects model when sample size is small.


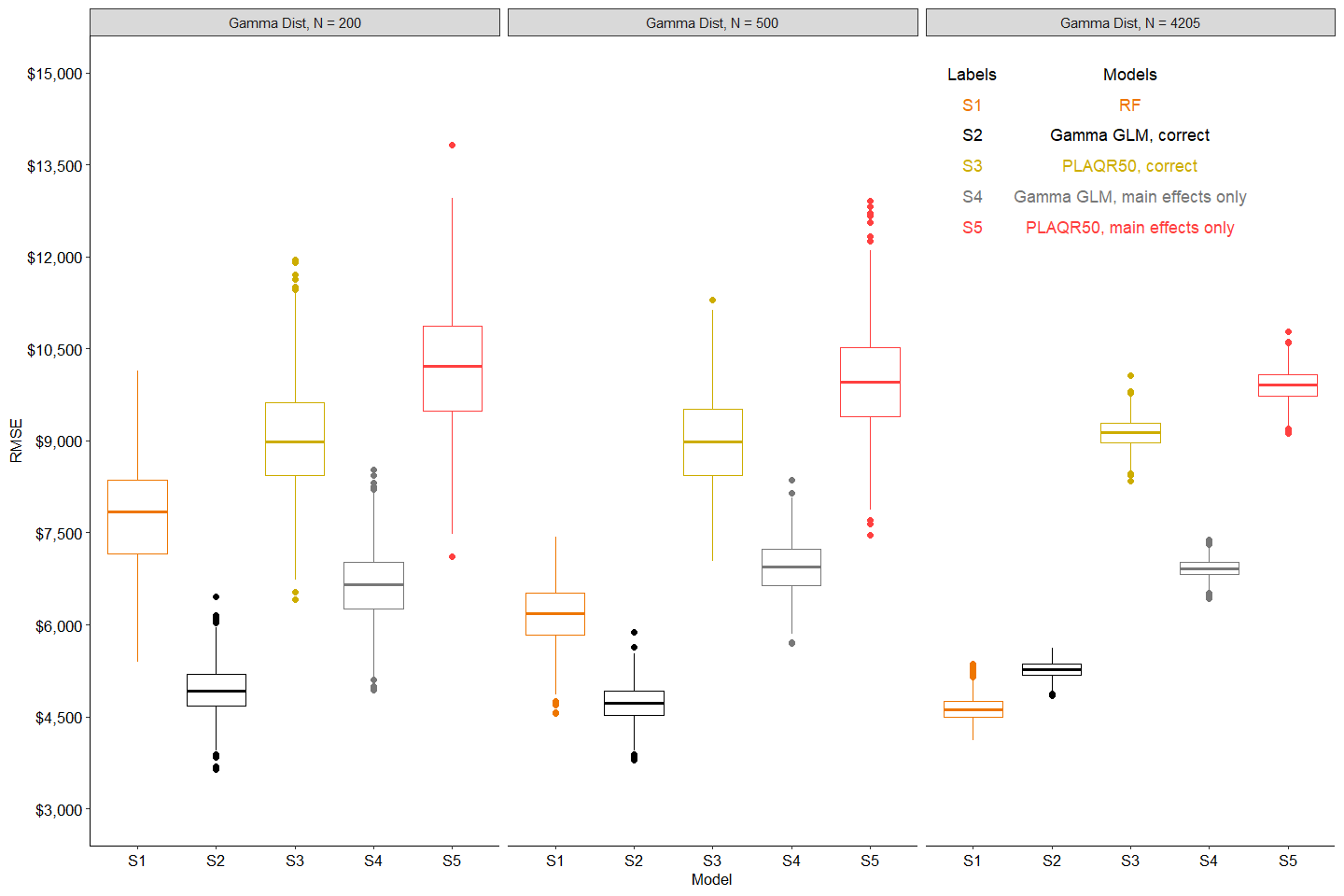


Figure S1A: Boxplots of RMSEs estimated from the five models for Gamma distribution with varied sample size. Boxplot of the RMSEs estimated from the 1000 bootstrapped samples are shown. RF, Gamma GLM correctly specified, PLAQR (estimating 50th percentile) correctly specified, main effects Gamma GLM and main effects PLAQR are compared.


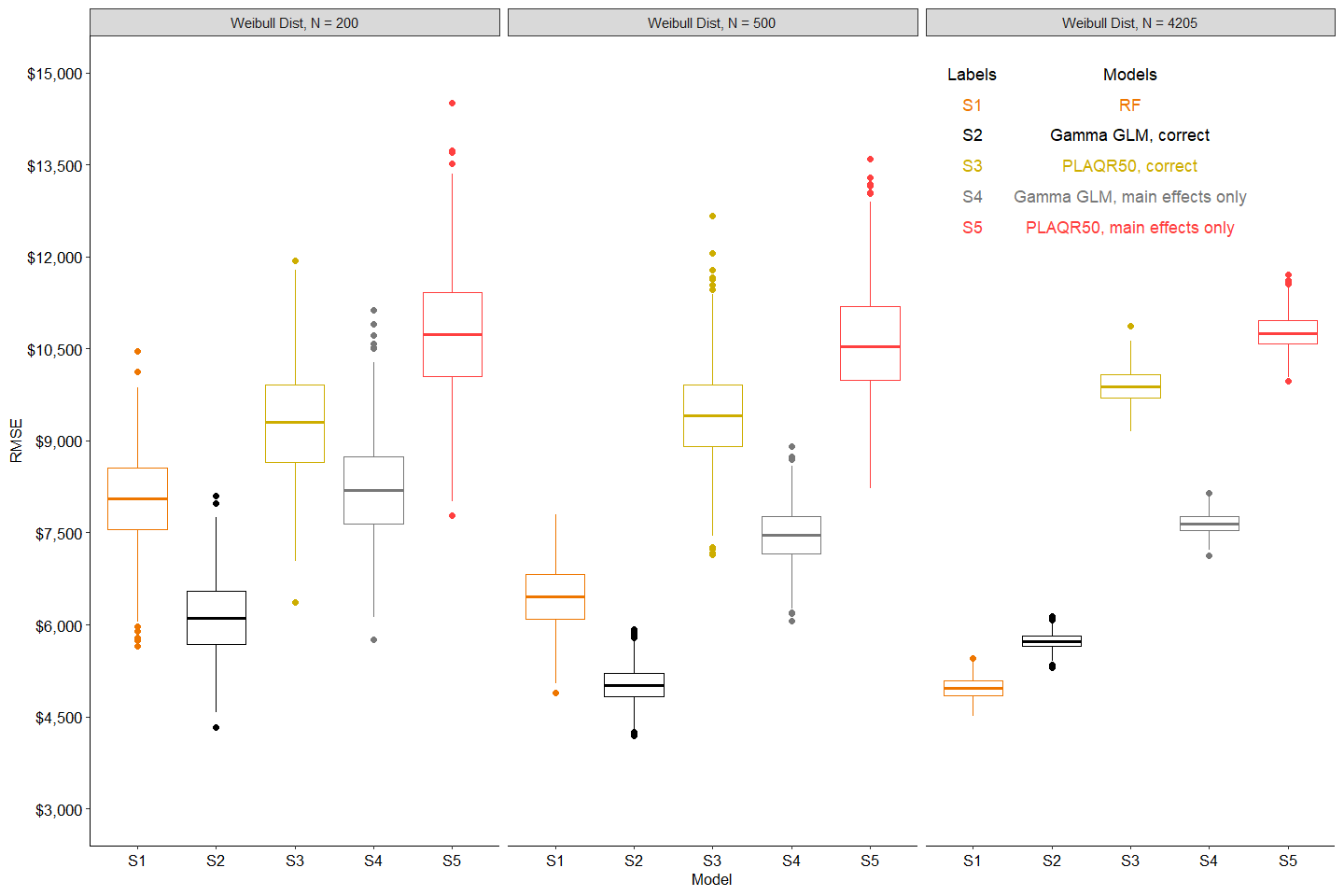


Figure S1B: Boxplots of RMSE estimated from the five models for Weibull distribution with varied sample size. Boxplot of the RMSEs estimated from the 1000 bootstrapped samples are shown. RF, Gamma GLM correctly specified, PLAQR (estimating 50th percentile) correctly specified, main effects Gamma GLM and main effects PLAQR are compared.


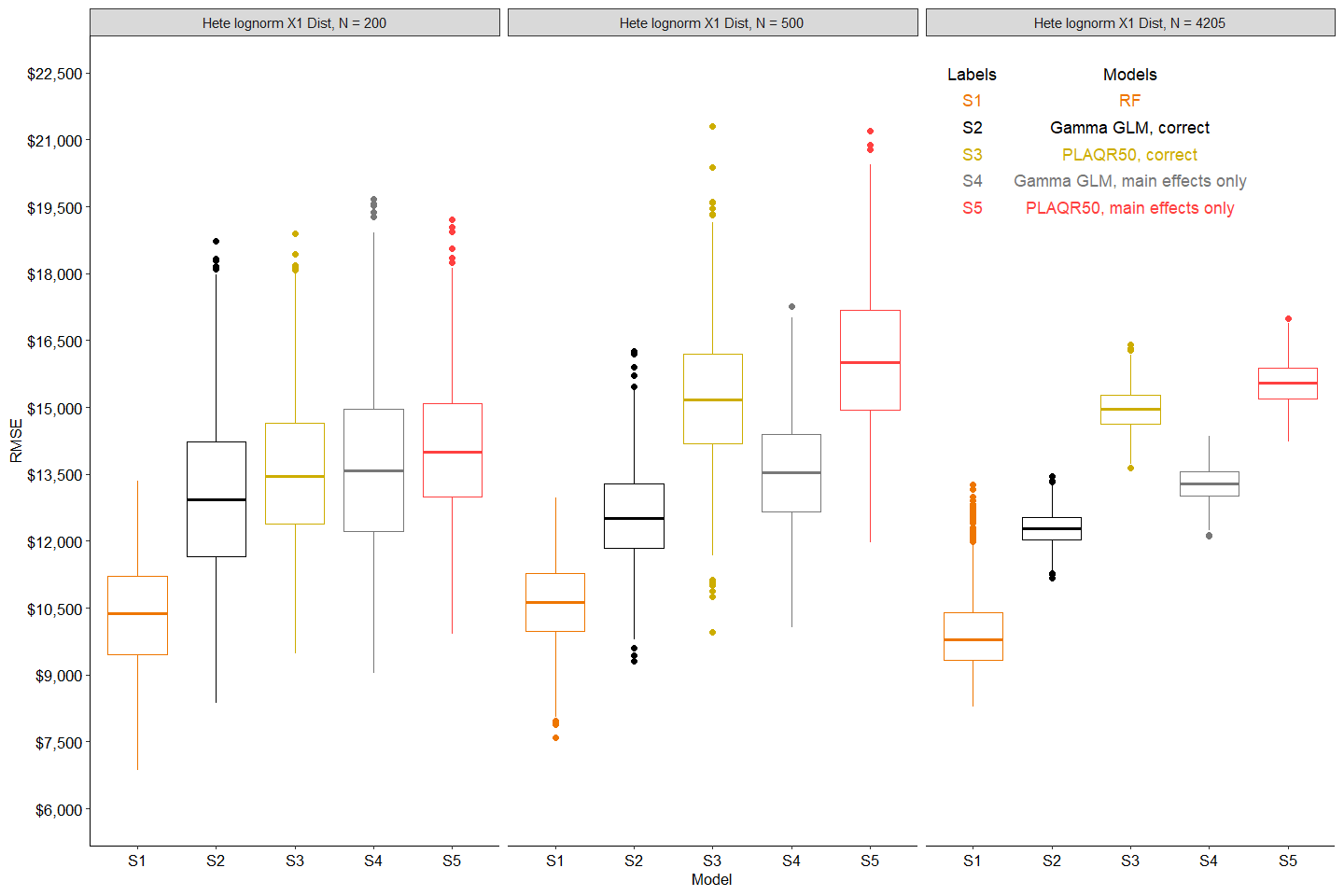


Figure S1C: Boxplots of RMSE estimated from the five models for heteroscedastic log-normal distribution with varied sample sizes. Boxplot of the RMSEs estimated from the 1000 bootstrapped samples are shown. RF, Gamma GLM correctly specified, PLAQR (estimating 50th percentile) correctly specified, main effects Gamma GLM and main effects PLAQR are compared.


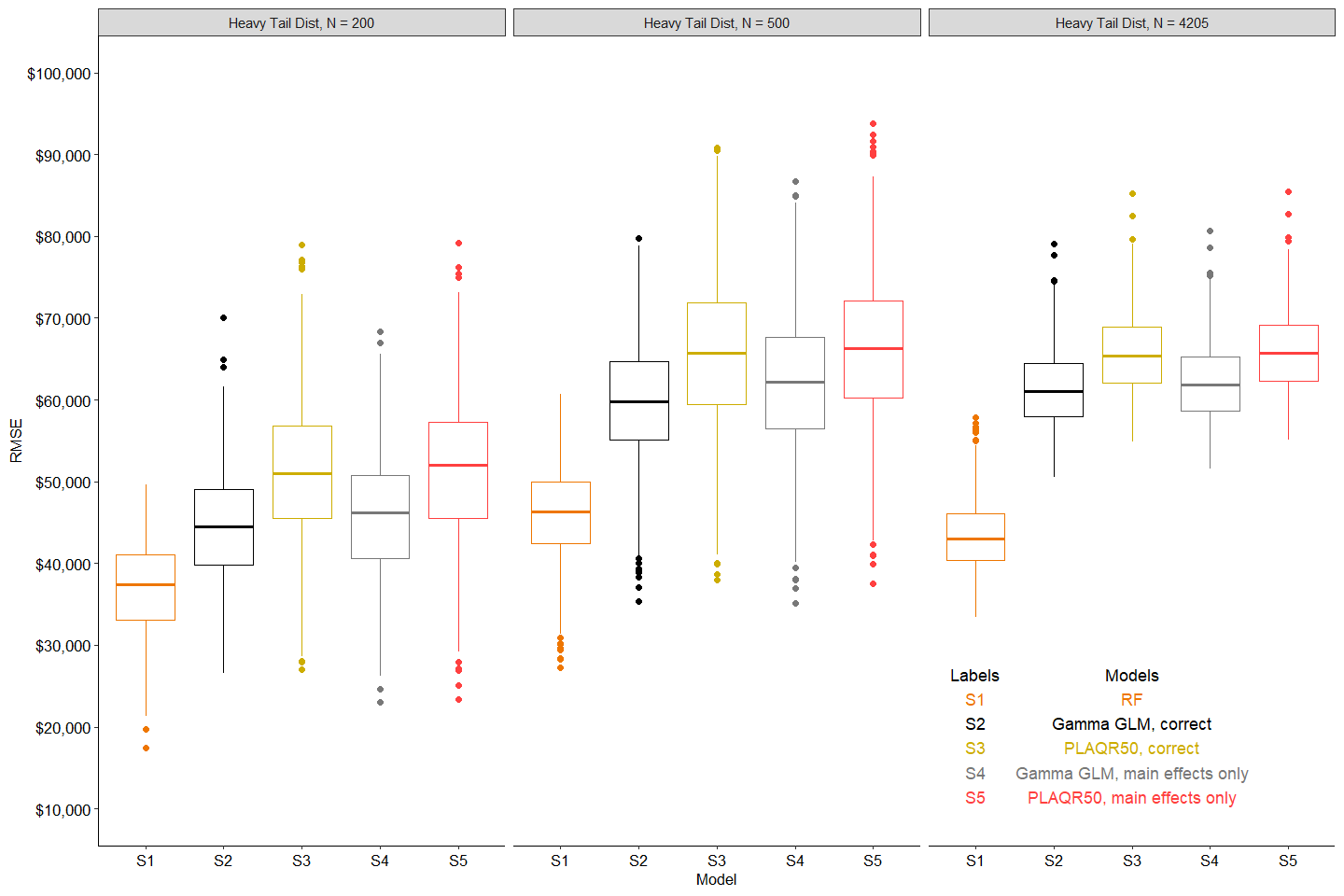


Figure S1D: Boxplots of RMSE estimated from the five models for heavy-tailed distribution with varied sample sizes. Boxplot from Gamma GLM with correct specification of interaction and non-linear terms for N = 200 was plotted based on the 286 converged models and boxplot from Gamma GLM with main effects only for the same scenario was plotted based on the 680 converged models. Boxplots from other scenarios were plotted based on the 1000 bootstrapped samples. RF, Gamma GLM correctly specified, PLAQR (estimating 50th percentile) correctly specified, main effects Gamma GLM and main effects PLAQR are compared.

***Section 2. Supplementary tables***

Table S1: Performance metrics of the three methods on the test data set, randomly split from the OCM data

|  | RMSE | MAPE | CA |
| --- | --- | --- | --- |
| Random forest | 8575.90 | 6280.76 | 64.20 |
| Gamma GLM | 18981.92 | 13502.21 | 38.43 |
| PLAQR | 18182.84 | 12713.84 | 8.54 |

Table S2: Summary statistics of the distributions of expenses observed in the OCM data and generated in the simulation data sets

|  | Median | Mean | Skewness | Kurtosis | 90th percentile |
| --- | --- | --- | --- | --- | --- |
| OCM data | 20551.6 | 27328.9 | 1 | 0.6 | 61884.9 |
| Gamma | 19378 | 27022.7 | 1.5 | 2.3 | 59057.5 |
| Weibull | 20497.8 | 28488.1 | 1.5 | 2.4 | 63108.1 |
| Hete log-normal | 19700.1 | 28652.9 | 2.8 | 21.5 | 62158.6 |
| Heavy tail | 18843.7 | 39368 | 5.7 | 53.2 | 91453.9 |
